# Supplementary material for: Information encoded in volumes and areas of dendritic spines is nearly maximal across mammalian brains
Source: Sci Rep. 2023 Dec 14;13:22207. doi: 10.1038/s41598-023-49321-9 (PMC10721930; doi:10.1038/s41598-023-49321-9)
Supplement: Supplementary file 1 — Supplementary Information. [file 41598_2023_49321_MOESM1_ESM.pdf]

Supplemental Information for:

Information encoded in volumes and areas of dendritic spines is nearly maximal across mammalian brains

Jan Karbowski[1,*], Paulina Urban[2,3,4]

[1] *Institute of Applied Mathematics and Mechanics, University of Warsaw, Warsaw, Poland;* [2] *Laboratory of Functional and Structural Genomics, Centre of New Technologies, University of Warsaw, Warsaw, Poland;* [3] *College of Inter-Faculty Individual Studies in Mathematics and Natural Sciences, University of Warsaw, Warsaw, Poland;* [4] *Laboratory of Databases and Business Analytics, National Information Processing Institute, National Research Institute, Warsaw, Poland.*

Supplementary Figures

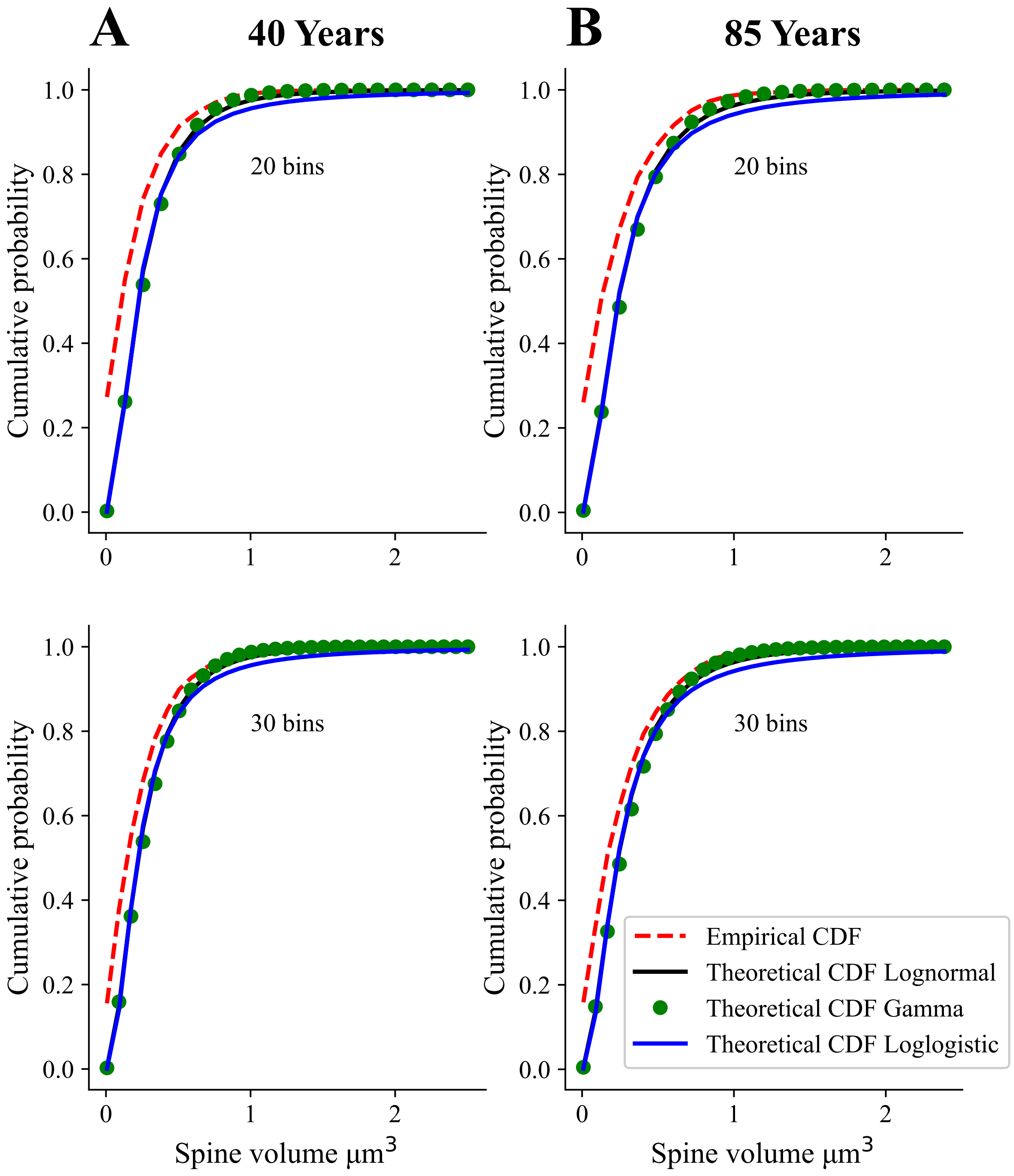
A
40 Years
B
85 Years
20 bins
30 bins
Cumulative probability
Spine volume μm3
Empirical CDF
Theoretical CDF Lognormal
Theoretical CDF Gamma
Theoretical CDF Loglogistic
0.0
0.2
0.4
0.6
0.8
1.0
0
1
2

Figure S1

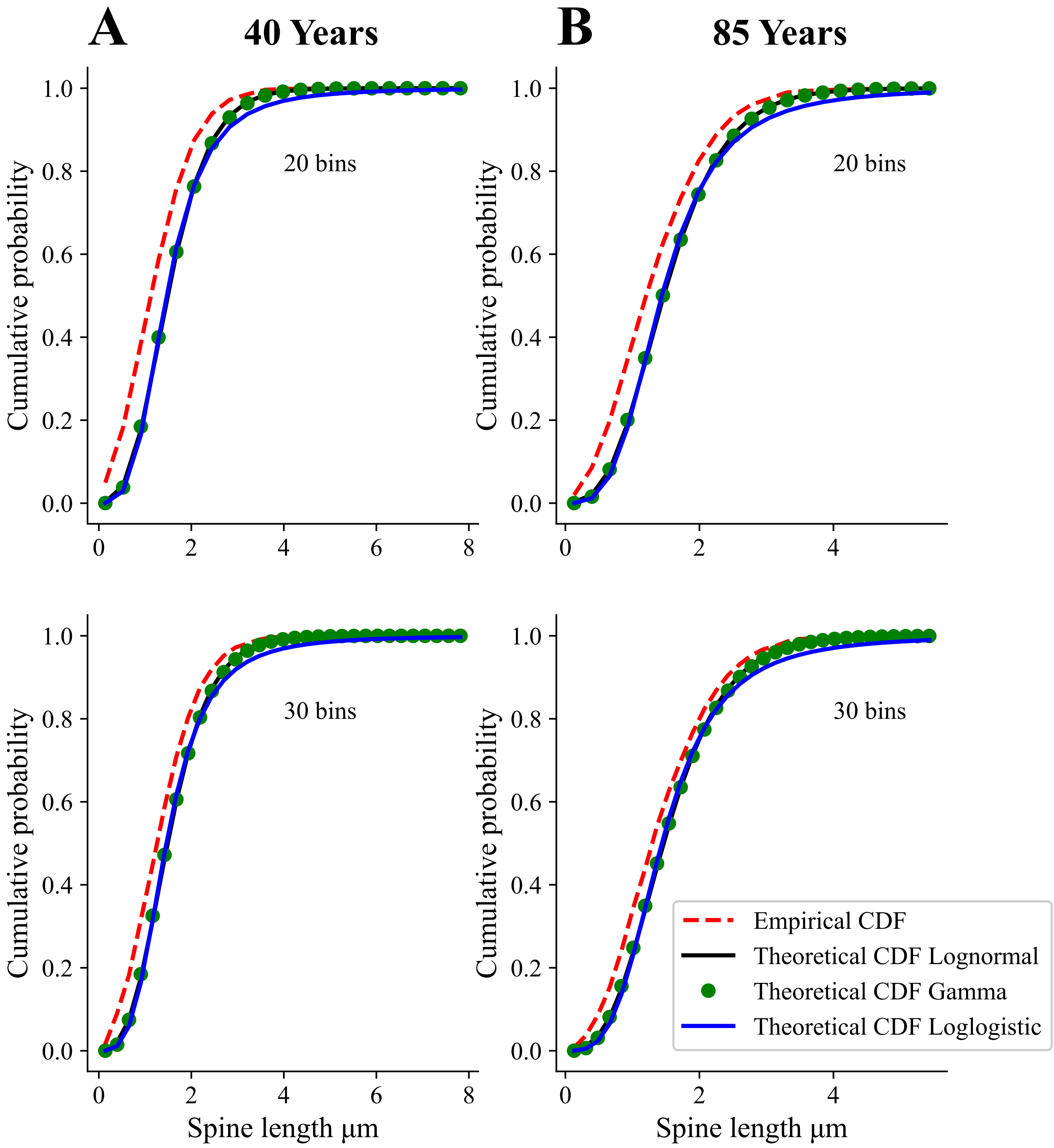

A
40 Years
B
85 Years
Cumulative probability
20 bins
30 bins
Spine length μm
Empirical CDF
Theoretical CDF Lognormal
Theoretical CDF Gamma
Theoretical CDF Loglogistic

Figure S2

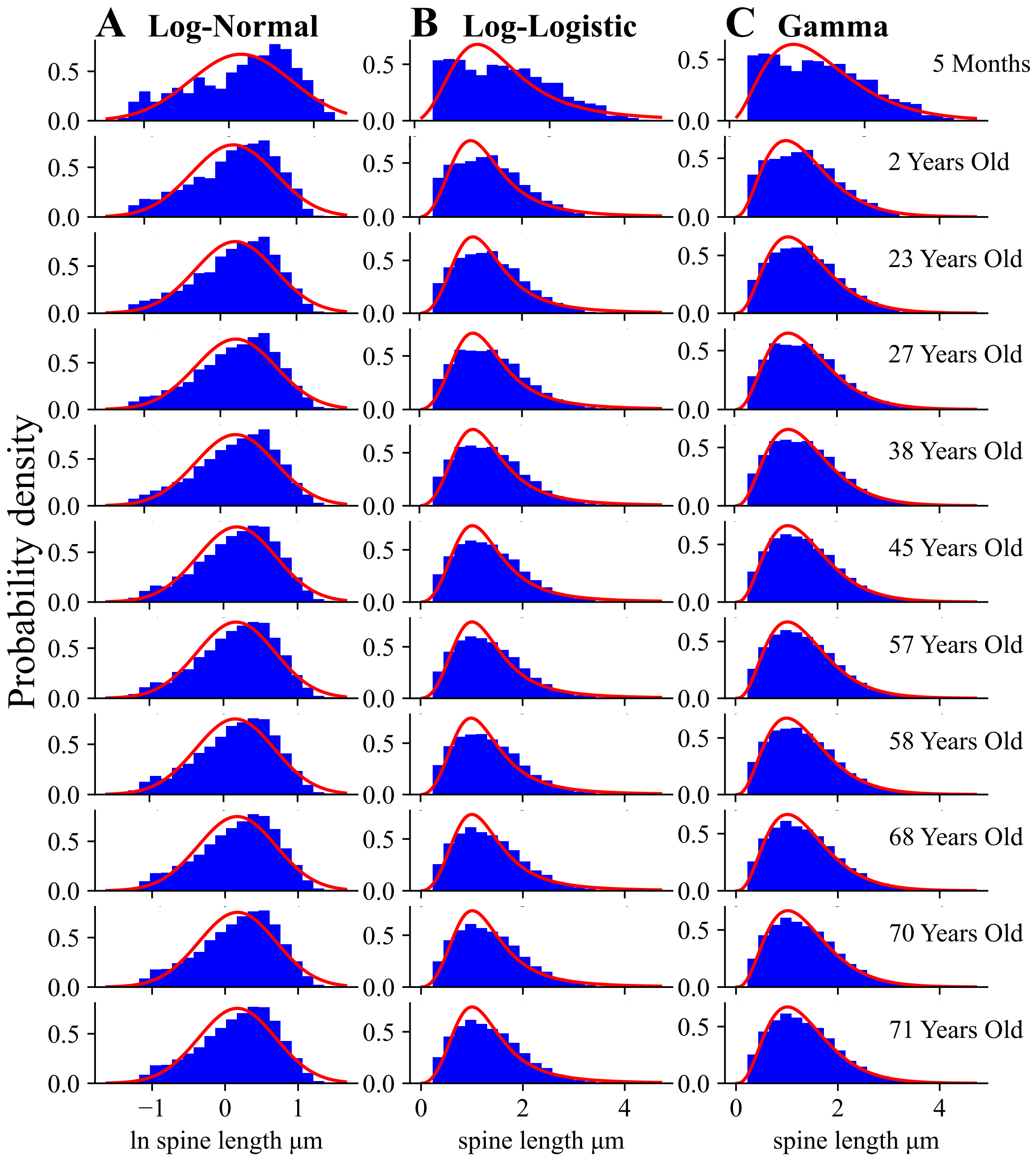
A Log-Normal
B Log-Logistic
C Gamma
5 Months
2 Years Old
23 Years Old
27 Years Old
38 Years Old
45 Years Old
57 Years Old
58 Years Old
68 Years Old
70 Years Old
71 Years Old
Probability density
0.0
0.5
−1
0
1
2
4
ln spine length μm
spine length μm
spine length μm

Figure S3

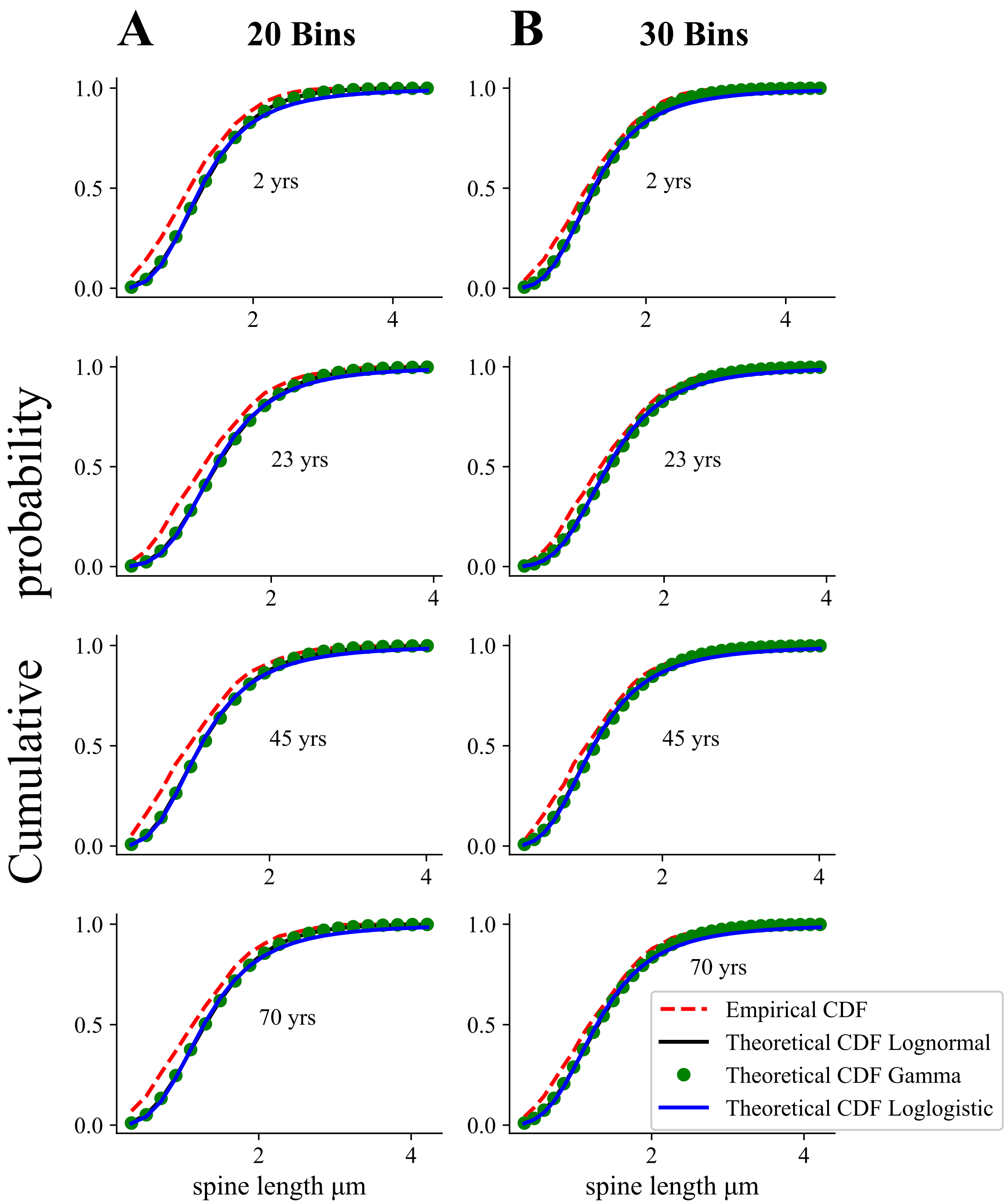
A
20 Bins
B
30 Bins
Cumulative probability
2 yrs
23 yrs
45 yrs
70 yrs
spine length µm
Empirical CDF
Theoretical CDF Lognormal
Theoretical CDF Gamma
Theoretical CDF Loglogistic

Figure S4

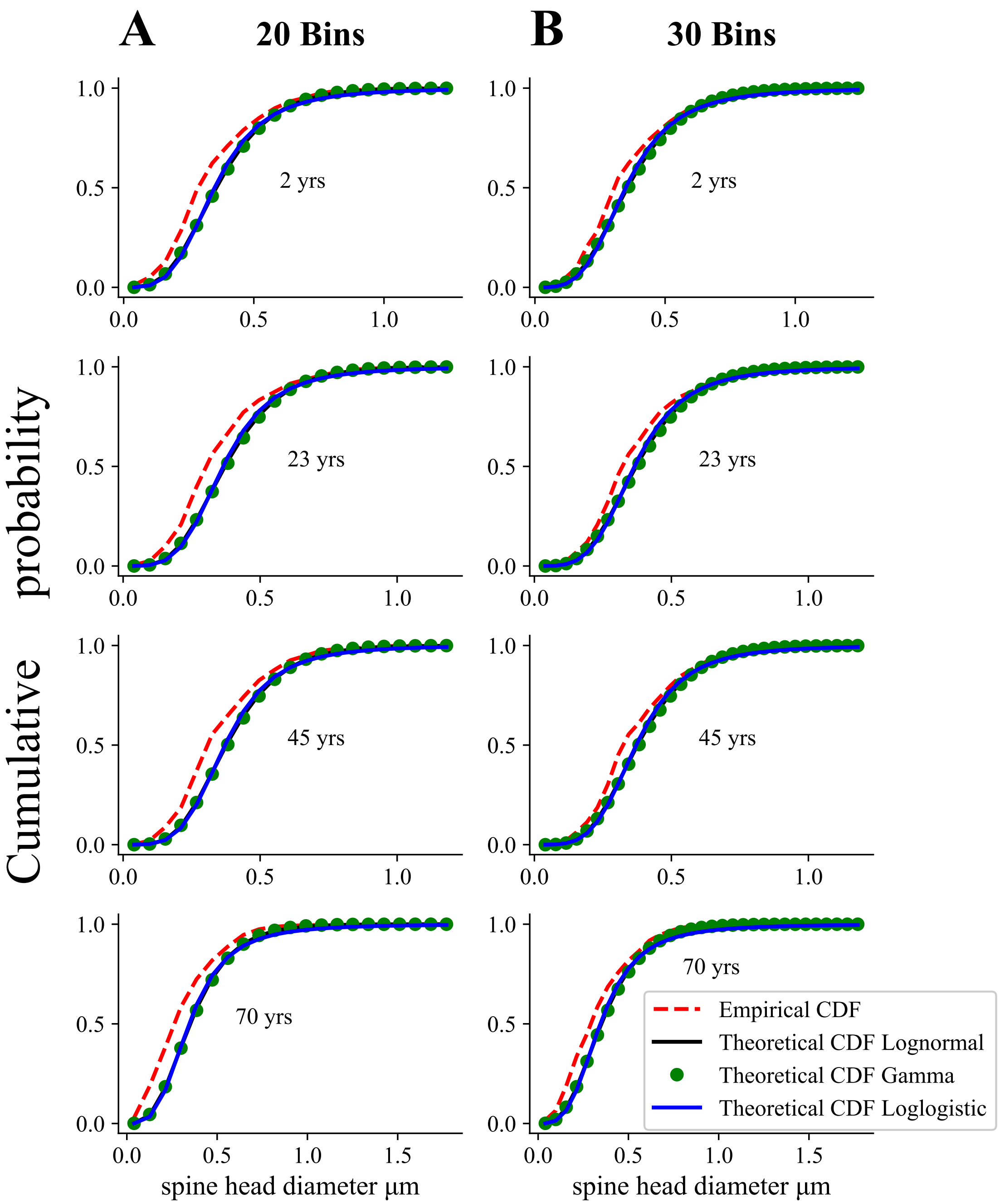
A
20 Bins
B
30 Bins
Cumulative probability
2 yrs
23 yrs
45 yrs
70 yrs
spine head diameter µm
Empirical CDF
Theoretical CDF Lognormal
Theoretical CDF Gamma
Theoretical CDF Loglogistic

Figure S5

Figure Captions

Fig. S1

Cumulative distribution function CDF of spine volume for empirical and theoretical distributions in cingulate cortex. Note that all three theoretical CDF are close to the empirical CDF, but gamma and lognormal are slightly better than loglogistic.

Fig. S2

Similar as in Fig. S1, but for spine length.

Fig. S3

Similarity of spine length distributions across human lifespan. Empirical data for human hippocampal spine length (rectangles; taken from Das et al (2019)) ranging from infancy (except 5 month old), through maturity, to senility look very similar. These data were fitted to three different distributions (solid lines). Mean values of the fitted parameters and corresponding 95% confidence intervals (in the brackets) are provided below.

A) Fitting parameters for lognormal: $\mu = 0.14$ CI=[0.10, 0.18], $\sigma = 0.59$ CI=[0.56, 0.61], (5 months); $\mu = 0.17$ CI=[0.14, 0.19], $\sigma = 0.55$ CI=[0.53, 0.56], (2 years); $\mu = 0.19$ CI=[0.17, 0.21], $\sigma = 0.52$ CI=[0.51, 0.53], (23 years); $\mu = 0.20$ CI=[0.18, 0.22], $\sigma = 0.53$ CI=[0.51, 0.54], (27 years); $\mu = 0.20$ CI=[0.18, 0.21], $\sigma = 0.52$ CI=[0.51, 0.53], (38 years); $\mu = 0.18$ CI=[0.16, 0.19], $\sigma = 0.53$ CI=[0.51, 0.54], (45 years); $\mu = 0.17$ CI=[0.15, 0.18], $\sigma = 0.52$ CI=[0.51, 0.53], (57 years); $\mu = 0.16$ CI=[0.14, 0.17], $\sigma = 0.53$ CI=[0.52, 0.54], (58 years); $\mu = 0.16$ CI=[0.15, 0.17], $\sigma = 0.53$ CI=[0.52, 0.54], (68 years); $\mu = 0.17$ CI=[0.16, 0.18], $\sigma = 0.53$ CI=[0.52, 0.54], (70 years); $\mu = 0.17$ CI=[0.15, 0.18], $\sigma = 0.53$ CI=[0.52, 0.54], (71 years).

B) Fitting parameters for loglogistic: $a = 1.20$ CI=[0.68, 1.71], $b = 2.86$ CI=[1.53, 4.19], (5

months); a = 1.23 CI=[0.85, 1.60], b = 3.13 CI=[2.09, 4.16], (2 years); a = 1.26 CI=[0.96, 1.55], b = 3.33 CI=[2.49, 4.17], (23 years); a = 1.26 CI=[1.03, 1.48], b = 3.23 CI=[2.60, 3.86], (27 years); a = 1.26 CI=[1.07, 1.45], b = 3.33 CI=[2.80, 3.86], (38 years); a = 1.23 CI=[1.08, 1.38], b = 3.23 CI=[2.80, 3.66], (45 years); a = 1.22 CI=[1.08, 1.35], b = 3.33 CI=[2.92, 3.73], (57 years); a = 1.21 CI=[1.08, 1.33], b = 3.23 CI=[2.85, 3.60], (58 years); a = 1.21 CI=[1.09, 1.32], b = 3.23 CI=[2.88, 3.57], (68 years); a = 1.22 CI=[1.10, 1.33], b = 3.23 CI=[2.90, 3.56], (70 years); a = 1.21 CI=[1.10, 1.32], b = 3.23 CI=[2.91, 3.54], (71 years).

C) Fitting parameters for gamma: α = 3.35 CI=[2.84, 3.86], β = 2.48 CI=[2.18, 2.91], (5 months); α = 3.84 CI=[3.07, 4.60], β = 2.83 CI=[2.48, 3.30], (2 years); α = 4.17 CI=[3.37, 4.96], β = 3.03 CI=[2.70, 3.46], (23 years); α = 4.07 CI=[3.50, 4.63], β = 2.93 CI=[2.67, 3.24], (27 years); α = 4.13 CI=[3.65, 4.60], β = 2.99 CI=[2.76, 3.26], (38 years); α = 4.06 CI=[3.70, 4.42], β = 2.99 CI=[2.80, 3.20], (45 years); α = 4.11 CI=[3.77, 4.45], β = 3.05 CI=[2.87, 3.25], (57 years); α = 4.03 CI=[3.73, 4.33], β = 3.03 CI=[2.86, 3.21], (58 years); α = 4.01 CI=[3.73, 4.28], β = 2.99 CI=[2.84, 3.16], (68 years); α = 4.07 CI=[3.79, 4.34], β = 3.02 CI=[2.87, 3.18], (70 years); α = 4.07 CI=[3.80, 4.33], β = 3.04 CI=[2.90, 3.20], (71 years).

Fig. S4

Cumulative distribution function CDF of spine length for empirical and theoretical distributions in hippocampus. Note a near overlap of all three theoretical CDF with the empirical CDF.

Fig. S5

Similar as in Fig. S4, but for spine head diameter.

Supplementary Tables

Table 1: (T1) Kolmogorov-Smirnov goodness of fit for spine volume and length: human cingulate cortex.

Bin no N_b	Spine Parameter	Age (yrs)	Kolmogorov-Smirnov distance D_{KS}		
			Lognormal	Loglogistic	Gamma
20					
	Volume	40	0.269*	0.272	0.274
	Volume	85	0.256*	0.259	0.256*
	Length	40	0.190	0.199	0.179*
	Length	85	0.133	0.140	0.122*
30					
	Volume	40	0.221	0.230	0.212*
	Volume	85	0.193	0.201	0.183*
	Length	40	0.127	0.137	0.117*
	Length	85	0.091	0.094	0.080*
412					
	Volume	40	0.026	0.034	0.020*
	Volume	85	0.033	0.037	0.019*
	Length	40	0.019*	0.026	0.020
	Length	85	0.018	0.029	0.014*

All fits to the three theoretical distributions are statistically significant at the level of $P = 0.05$ (regardless of the number of bins N_b), since the Kolmogorov-Smirnov distance D_{KS} is always smaller than the critical distance D_{cr}, which is 0.294 for $N_b = 20$, 0.240 for $N_b = 30$, and 0.067 for $N_b = 412$ (Keeping 1995). The asterisk indicates the smallest KS distance, and hence the best theoretical distribution from the chosen three.

Table 2: (T2) Kolmogorov-Smirnov goodness of fit for spine length and head diameter: human hippocampus.

Bin N_b	Spine Param.	Distrib.	Kolmogorov-Smirnov distance D_{KS} Age (yrs)										
			$\frac{5}{12}$	2	23	27	38	45	57	58	68	70	71
20	Length	Lognor.	0.128	0.126	0.123	0.127	0.127	0.127	0.129	0.131	0.128	0.127	0.128
		Loglog.	0.135	0.134	0.134	0.138	0.138	0.138	0.139	0.141	0.138	0.138	0.139
		Gamma	0.123*	0.118*	0.117*	0.122*	0.122*	0.122*	0.124*	0.127*	0.123*	0.122*	0.124*
20	H. diam.	Lognor.	0.120	0.131	0.135	0.159	0.162	0.163	0.165	0.169	0.166	0.198	0.196
		Loglog.	0.119	0.130	0.135	0.157*	0.169	0.171	0.173	0.176	0.174	0.193*	0.191
		Gamma	0.116*	0.128*	0.133*	0.157*	0.159*	0.159*	0.161*	0.166*	0.163*	0.201	0.198*
30	Length	Lognor.	0.099	0.099	0.091	0.092	0.092	0.090	0.090	0.092	0.093	0.092	0.093
		Loglog.	0.107	0.108	0.102	0.100	0.099	0.099	0.100	0.102	0.103	0.103	0.103
		Gamma	0.094*	0.092*	0.085*	0.084*	0.085*	0.085*	0.085*	0.087*	0.088*	0.088*	0.088*
30	H. diam.	Lognor.	0.087	0.098	0.098	0.113	0.123	0.126	0.129	0.131	0.128	0.135*	0.133*
		Loglog.	0.080*	0.091*	0.088*	0.112	0.113*	0.116*	0.119*	0.120*	0.117*	0.138	0.137
		Gamma	0.081	0.098	0.100	0.110*	0.127	0.130	0.132	0.135	0.132	0.140	0.138
256	Length	Lognor.	0.088	0.070	0.063	0.061	0.058	0.057	0.055	0.056	0.055	0.055	0.054
		Loglog.	0.069*	0.049	0.038	0.038	0.038	0.035	0.035	0.036*	0.036	0.037	0.036
		Gamma	0.077	0.046*	0.035*	0.036*	0.034*	0.035*	0.033*	0.036*	0.034*	0.033*	0.034*
100	H. diam.	Lognor.	0.062	0.057	0.055	0.055	0.046	0.047	0.047	0.048	0.047	0.045	0.044
		Loglog.	0.032*	0.038*	0.038*	0.035*	0.031*	0.034*	0.035*	0.034*	0.034*	0.031*	0.031*
		Gamma	0.033	0.054	0.055	0.050	0.048	0.050	0.051	0.053	0.051	0.049	0.046

All fits to the three theoretical distributions are statistically significant at the level of $P = 0.05$ (regardless of the number of bins N_b), since the Kolmogorov-Smirnov distance D_{KS} is always smaller than the critical distance D_{cr}, which is 0.294 for $N_b = 20$, 0.240 for $N_b = 30$, and 0.067 for $N_b = 412$ (Keeping 1995). The asterisk indicates the smallest KS distance, and hence the best theoretical distribution from the chosen three.

Code for data analysis: KS goodness of fit.

```
# -*- coding: utf-8 -*-
from pylab import *
import numpy as np
import matplotlib.pyplot as plt
import scipy.stats as st
import random
import math
import csv
import pandas as pd
import seaborn as sns
from scipy.optimize import curve_fit
from collections import Counter
import warnings
import time
import matplotlib
matplotlib.rc('xtick', labelsize=10)
matplotlib.rc('ytick', labelsize=10)
import warnings
warnings.filterwarnings('ignore')

# reading data
df1 = pd.read_excel('human_benevides.xls',sheet_name='spine length')
pacjent = df1['C40']
#pacjent = df1['C85']
newlist = [e for e in pacjent if math.isnan(e) == False]

##LOG-NORMAL
mean, std = st.norm.fit(np.log(newlist))
a,b,c = st.lognorm.fit(newlist)

## gamma
fit_alpha1, fit_loc1, fit_beta1 = st.gamma.fit(newlist,floc=0)

## loglogistic
fit_alpha2, fit_loc2, fit_beta2 = st.fisk.fit(newlist,floc=0)

# calculate ecdf when number of bins are equal to maximal number for data set
def ecdf2(sample):
 sample = np.atleast_1d(sample)
 quantiles, counts = np.unique(sample, return_counts=True)
 cumprob = np.cumsum(counts).astype(np.double) / sample.size
 return quantiles, cumprob

# calculate ecdf when number of bins equal to 20 or 30
def ecdf(sample):
 sample = np.atleast_1d(sample)
 values, binki = np.histogram(sample, bins=30, normed=True)
 pdf = values / sum(values)
 data_cum = np.cumsum(pdf)
 return binki, data_cum
```

```
n1 = st.lognorm(a,b,c)
n2 = st.gamma(fit_alpha1, loc=fit_loc1, scale= fit_beta1)
n3 = st.fisk(fit_alpha2, loc=fit_loc2, scale= fit_beta2)

# # compute the ECDF of the samples
qe, pe = ecdf2(newlist)

# evaluate the theoretical CDF over the same range
q1 = np.linspace(qe[0], qe[-1], len(qe))
p1 = n1.cdf(q1)
q2 = np.linspace(qe[0], qe[-1], len(qe))
p2 = n2.cdf(q2)
q3 = np.linspace(qe[0], qe[-1], len(qe))
p3 = n3.cdf(q3)

cdfs = ['norm','fisk','gamma']
data_sample = newlist

# calculate "goodness of fit"
for cdf in cdfs:
 parameters = eval("st."+cdf+".fit(data_sample)")
 D, p = st.kstest(data_sample, cdf, args=parameters, N = 30)
 print (cdf.ljust(16) + ("p: "+str('{0:.10f}'.format(p)).ljust(40)+"D: "+str('{0:.10f}'.format(D))))
```
